# Multimaterial magnetically assisted 3D printing of composite materials

**DOI:** 10.1038/ncomms9643

**Published:** 2015-10-23

**Authors:** Dimitri Kokkinis, Manuel Schaffner, André R. Studart

**Affiliations:** 1 Complex Materials, Department of Materials, ETH Zürich, Zürich 8093, Switzerland

## Abstract

3D printing has become commonplace for the manufacturing of objects with unusual geometries. Recent developments that enabled printing of multiple materials indicate that the technology can potentially offer a much wider design space beyond unusual shaping. Here we show that a new dimension in this design space can be exploited through the control of the orientation of anisotropic particles used as building blocks during a direct ink-writing process. Particle orientation control is demonstrated by applying low magnetic fields on deposited inks pre-loaded with magnetized stiff platelets. Multimaterial dispensers and a two-component mixing unit provide additional control over the local composition of the printed material. The five-dimensional design space covered by the proposed multimaterial magnetically assisted 3D printing platform (MM-3D printing) opens the way towards the manufacturing of functional heterogeneous materials with exquisite microstructural features thus far only accessible by biological materials grown in nature.

## Abstract (web summary)

Endowing composite materials with spatially discrete mechanical behaviours is possible by varying the internal concentration and arrangement of particles. Here, the authors demonstrate a 3D magnetic printing technique which enables the fabrication of materials with intricate internal designs.

## Graphical abstract

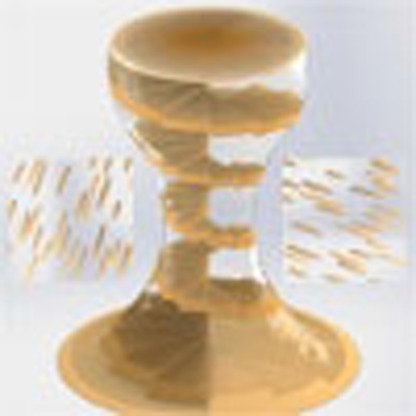

The unprecedented three-dimensional (3D) shaping capabilities of additive manufacturing technologies have long been exploited for rapid prototyping^1^,^2^ and more recently have influenced fields as diverse as medicine^3^, anthropology^4^ and design^5^. The possibility of combining multiple materials in a single geometry is an even more powerful asset of this fabrication approach that adds on an extra dimension in the available design space.

Multimaterial additive manufacturing has enabled the implementation of enticing functionalities into 3D objects^6^,^7^,^8^,^9^,^10^,^11^,^12^,^13^,^14^. For example, inks loaded with cathode and anode materials have allowed for direct writing of lithium-ion microbatteries with high areal energy and power densities^7^. Combining cells, biocompatible hydrogels and inorganic particles in the printing process has been extensively explored for the fabrication of advanced scaffolds for tissue regeneration^8^,^9^. Flexible, protective armors inspired by the architecture of fish scales have also been recently produced through 3D printing^10^. Programmable objects that can dynamically change their geometry have been printed with elastomeric, glassy, thermoresponsive and shape memory polymers^6^,^11^,^12^,^13^,^15^,^16^,^17^.

Although synthetic processes are far less elaborate and cover a much narrower range of length scales as compared with biologically mediated assembly, the deposition of multiple materials that incorporate new functionalities in 3D printed parts resembles, to some extent, the approach used by living cells to grow biological materials in nature. Alike printing heads, cells in the connective tissues of animals and plants lay down extracellular material (ECM) with locally tunable composition in a layer-by-layer manner. Despite such similarity, a major difference between ECM deposition in biology and man-devised printing processes with regards to the multimaterial aspects is the final deposited material. The ECM often comprises modular, anisotropic constituents such as fibrils, fibres and mineral particles, which are locally aligned in specific directions at different concentrations to generate highly textured composite materials^18^. Here texture refers to the deliberate alignment of anisotropic building blocks to enhance target properties in particular orientations. Importantly, independent local composition and texture control provide living cells with at least two additional degrees of freedom as compared with regular 3D printing processes, leading to static and dynamic properties that find no counterparts in artificial systems^19^,^20^,^21^. Although recent studies have shown that it is possible to print composite inks containing discontinuous anisotropic fibres^22^,^23^, deliberate control of the local orientation of the deposited particles has not yet been accomplished.

Inspired by the elaborate heterogeneous architectures found in natural materials, we propose an additive manufacturing route that enables programming and fabrication of synthetic microstructures within a five-dimensional design space. Besides the 3D shaping capabilities of additive manufacturing, such a design space also includes local control of composition (+1D) and particle orientation (+1D). The creation of biomimetic materials through additive manufacturing is the realization of a long sought-after vision^24^ that, except from a few recent examples^6^,^10^,^17^,^23^, has been hampered by the lack of easily accessible inks and manufacturing tools. In this report, we first introduce the printing platform developed to enable 5D programmability. This is followed by a description of the composition and rheology of inks formulated to allow for the fabrication of distortion-free 3D shapes. The dynamics of the magnetically assisted process used to achieve particle orientation control is then discussed. Finally, we exploit the proposed multimaterial magnetically assisted 3D printing system (MM-3D printing) to produce exemplary heterogeneous composites with unparalleled microstructural features and multifunctional shape-changing soft devices for adhesive-free mechanical fastening. We highlight that the development of this new printing platform and its use to fabricate 3D self-shaping objects strongly relied on a fundamental understanding of (i) the relation between ink rheology and the shape distortion of filaments, (ii) the dynamics of the alignment of anisotropic particles under an external magnetic field and (iii) the correlation between shape changes in swelling objects and the geometry and properties of their constituent materials.

## Results

### MM-3D printing set-up and ink formulation

**Figure 1:**
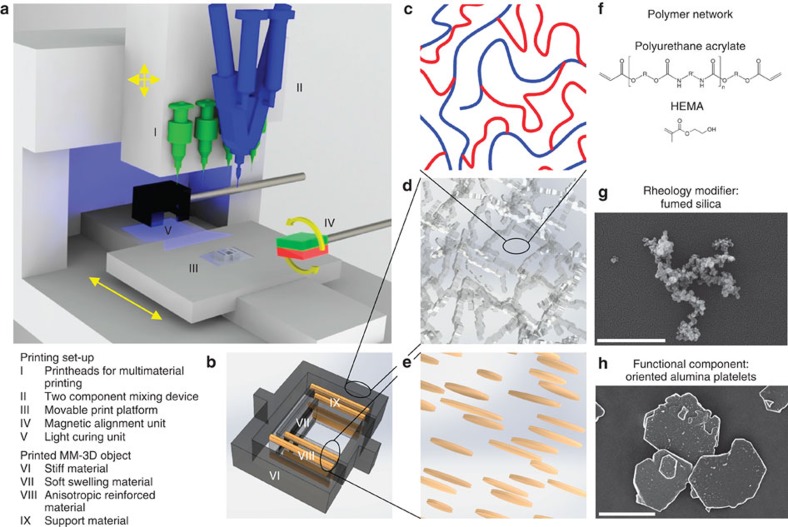
Schematics of the MM-3D printing platform for the creation of heterogeneous composites. (**a**) Direct ink-writing hardware equipped with multiple dispensers (I), a mixing unit (II), movable head and table (III), a magnet (IV) and a curing unit (V). (**b**) Example of MM-3D printed object designed to change shape on external stimulus. Different inks were formulated to results in (VI) a stiff material, (VII) a soft swelling material, (VIII) an anisotropic reinforced material, and (IX) a support material. (**c**–**e**) Illustrations of typical ink constituents. (**c**) Oligomers and monomers that form the base material and are crosslinked after deposition to generate a polymer network. (**d**) FS nanoparticles that percolate throughout the resin. (**e**) Anisotropic particles oriented by the external magnetic field. (**f**) chemical structures of typical ink monomers/oligomers. (**g**) SEM image of fumed silica particles. Scale bar, 500 nm. (**h**) SEM image of alumina platelets. Scale bar, 10 μm.

5D programmability is achieved by utilizing inks consisting of magnetically responsive anisotropic stiff particles suspended in a light-sensitive liquid resin of tunable composition. Such inks are deposited using a direct-writing approach in a commercial 3D printer, customized to enable particle alignment using a low-cost neodymium magnet (Fig. 1). The printer is equipped with four independently addressable syringes that can be charged with inks with different formulations. A two-component mixing and dispensing unit is also integrated into the printer to enable a gradual change in the ink composition during the printing process. Loading the two-component dispenser and the individual syringes with inks containing different initial particle contents and resin compositions allows for local control over the relative concentrations of building blocks in the printed layers. The initially non-magnetic particles are made magnetically responsive via the adsorption of iron oxide nanoparticles on their surface before the formulation of the ink^25^. Control of the orientation of anisotropic particles is possible by applying a low external magnetic field to the printed layer before consolidation of the continuous fluid phase by light. To gain spatial control of the local orientation of particles in a printed layer, we use masks of desired patterns during the curing process.

A crucial aspect for the realization of the MM-3D printing process is the formulation of inks that can provide the desired particle orientation, composition and 3D shape control. This requires inks with distinct flow responses that range from pure Newtonian for the orientation control to viscoelastic for the shape control. Such a wide spectrum was achieved by incorporating fumed silica (FS) particles of primary particle size of 5–30 nm as rheological modifier in the initial resin solution (Fig. 1d,g). To impart elasticity to the ink, the FS should form a percolating network when incorporated into the resin solution. This was accomplished by adding hydrophobic FS to more polar resins or, conversely, hydrophilic FS to less polar formulations. The ink formulations contained the following main components: polyurethane acrylate (PUA) oligomers, reactive diluents, photoinitiator, rheology modifier and modified alumina platelets as the texturable particles (Fig. 1c–h). The concentrations of the individual components of the inks were varied to fulfil the specific functionalities desired in each of the printed objects (Supplementary Tables 1 and 2). PUA oligomers that result in either soft or hard polymers after polymerization were used in different proportions as base constituents of all the inks. The reactive diluents were further added to change the mechanical properties of the inks after consolidation.

### Ink rheology and magnetic alignment of particles

**Figure 2:**
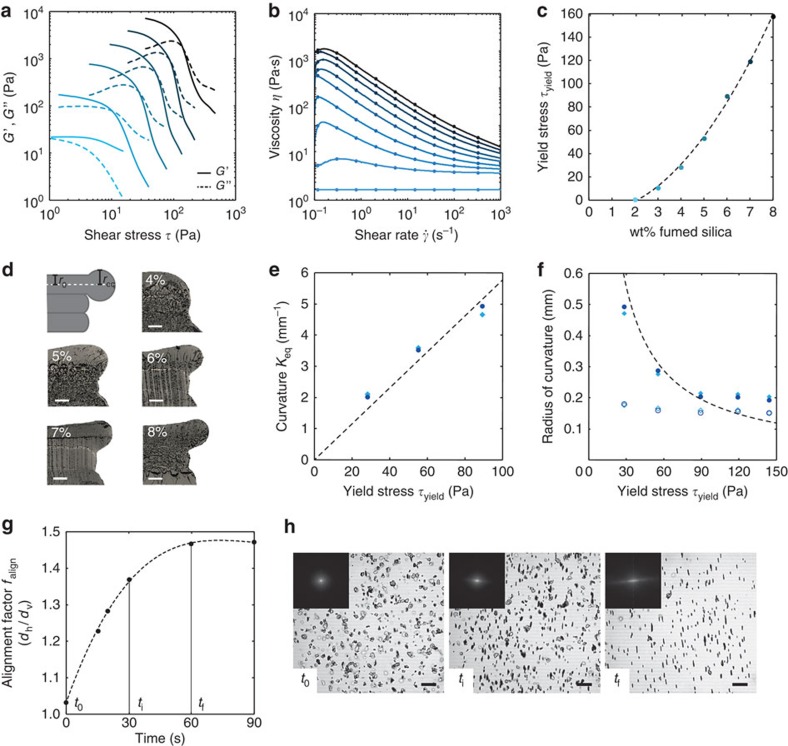
Rheology, shape retention and platelet alignment dynamics of the investigated composite inks. (**a**,**b**) Rheological data obtained under (**a**) oscillatory and (**b**) steady-shear conditions for inks with increasing concentration of FS: 2, 3, 4, 5, 6 and 8 wt. % FS in **a** and 0, 1, 2, 3, 4, 5, 6, 7 and 8 wt. % FS in **b**. The FS content increases from light to dark blue. (**c**) Effect of the FS content on the yield stress of the ink. (**d**–**f**) Analysis of shape distortion of inks with different FS concentrations. Micrographs in **d** exhibit cross-sections of three-layered films printed with inks containing increasing FS concentrations. An overhang was deliberately created here by offsetting the top layer relative to the others. The equilibrium radius of curvature (*r*_eq_) was measured at the overhang, while the initial radius (*r*_0_) was assumed to be half of the thickness of the layer. Scale bar, 200 μm. Graph **e** shows the direct correlation between the yield stress and the equilibrium curvature at low FS concentrations (4–6 wt%), when Δ*P*>*τ*_y_. A surface tension of 0.017 N m^−1^ was estimated based on the linear fit shown in this graph. Graph **f** shows the radius of curvature of the overhang as a function of the yield stress of the ink. Unfilled and filled symbols represent initial and equilibrium radii for 200-μm (diamonds) and 400-μm offsets (spheres), respectively. The dotted line indicates the equilibrium radius of curvature calculated from the experimentally obtained surface tension. (**g**,**h**) Alignment dynamics of platelets in the presence of a rotating magnetic field. Graph **g** shows the increase in the degree of platelet alignment as a function of time. Micrographs in **h** depict snapshots of the platelet suspensions at three different time points. Scale bar, 50 μm. The insets display FFTs of the optical images.

The effect of the addition of FS on the rheological behaviour of an exemplary resin composition was studied through oscillatory and steady-state measurements. From the rheological data obtained, we quantified the steady-state viscosity and the yield stress of the inks as depicted in Fig. 2a–c. The apparent yield stress of the ink (*τ*_y_) is taken here as the crossover between the storage and loss moduli measured in oscillatory rheological experiments. In the absence of FS, the base resin exhibits a Newtonian response with a viscosity of 0.2 Pa s, which is convenient for the alignment of anisotropic microparticles using low magnetic fields. On the addition of increasing amounts of FS particles to this base resin, the rheological response of the fluid changes from purely Newtonian to a viscoelastic behaviour with a well-defined apparent yield stress (Fig. 2a). Increasing the FS content from 2 to 8 wt% allows us to increase the yield stress and low-shear viscosity of the resin solution by two orders of magnitude (Fig. 2c). The viscoelastic nature of inks with high FS concentrations is crucial to prevent distortion of printed objects. Because the rheological modifier does not require a chemical reaction to thicken the fluid, many different resins including, for example, epoxies, silicones and acrylics were successfully printed using FS in the formulation. Thus, the proposed ink system provides a powerful platform with a broad palette of material's choice for the MM-3D printing of objects with orientation, composition and shape control.

To determine the yield stress needed to prevent shape distortion of objects with 3D complex geometries, we printed simple three-layered films with increasing concentrations of the rheological modifier and tracked possible changes in the top layer geometry after a fixed period of time (Fig. 2d). Distortions are typically driven by capillary forces that work to reduce the free energy of surfaces through a decrease in the local curvature of sharp printed edges and overhangs. To meet the conditions encountered in a standard printing process, the top layers were printed with representative overhangs offset by 200 or 400 μm on top of two existing layers. Shape distortion at the edge of the overhang was clearly observed for the inks with low apparent yield stress obtained at FS concentrations equal or below 5 wt% (Fig. 2d). Importantly, distortions were eliminated when the apparent yield stress imparted by the modifier was sufficiently high to prevent the action of capillary forces. For the acrylate-based ink investigated in this study, this condition was reached at a FS content of 6 wt% (Fig. 2d).

Understanding the interplay between capillary and elastic forces acting on the printed inks provides useful guidelines for the fabrication of complex-shaped objects free of distortion. Assuming that local shape distortion occurs when the Laplace capillary pressure (Δ*P*) across the surface of a layer edge overhang with initial radius *r*_i_ and curvature *κ*_i_ (1/*r*_i_) is higher than the apparent yield stress of the ink (*τ*_y_), one should expect the layer edge to deform to an equilibrium radius *r*_eq_ and curvature *κ*_eq_ (1/*r*_eq_) that varies according to the simple relation: *κ*_eq_=*τ*_y_/γ, where *γ* is the surface tension of the ink. Experimental data obtained for ink formulations with different FS concentrations confirm this linear dependence of the equilibrium curvature on the yield stress when Δ*P*>*τ*_y_ (Fig. 2e). Linear fitting of the experimental points leads to a surface tension *γ* of 0.017 N m^−1^. This estimate is close to the value of 0.019 N m^−1^ determined in independent surface tension measurements using the pendant drop technique, confirming that the proposed correlation effectively captures the capillarity-driven mechanism governing local shape distortion of the printed filaments. Inks with sufficiently high FS concentration (≥6 wt%) exhibit a yield stress higher than the capillary pressure (*τ*_y_>Δ*P*), which prevents excessive distortion of the printed material. In this case, an increase of 33–35% in the initial radius of curvature of the layer edge was consistently observed (Fig. 2f), probably because of slight relaxation of the overhanging viscoelastic material. On the basis of this analysis, we can predict the minimum FS concentration and yield stress required to prevent shape distortion of layer edge overhangs with different radii of curvature. This is shown in Fig. 2f for inks exhibiting a surface tension of 0.017 N m^−1^ (dashed line). In this case, we predict, for example, that the ink with the highest FS content of 8 wt% (*τ*_y_=160 Pa) can potentially enable printing of undistorted layers with edge radii approaching 100 μm. Taking into account the observed relaxation effect, this would require the deposition of lines with an initial radius of curvature at least 35% smaller than this value.

Besides the formation of stable edge overhangs, another crucial requirement of the MM-3D printing process is to ensure the alignment of anisotropic particles in the presence of an external magnetic field. According to a previous study^25^, the alignment of ultrahigh magnetically responsive platelets in Newtonian fluids with viscosity below a few Pa.s should be possible using low magnetic field strengths on the order of 1–10 mT. For the printing process, it is important that such an alignment occurs within short timescales to reduce the overall manufacturing time. To assess the dynamics of platelet alignment in our inks, we used an optical microscope to image the response of ultrahigh magnetically responsive platelets when subjected to a typical magnetic field of 40 mT rotating at 8.3 Hz (Fig. 2g,h). The magnetized platelets were suspended at a concentration of 1 wt% in a representative solution of monomers exhibiting a Newtonian viscosity of 1.15 Pa.s. By setting the frequency to 8.3 Hz, we ensured that the drag forces exerted on the platelets were sufficiently high to promote their biaxial alignment within the plane of the rotating field^26^.

The alignment of platelets was analysed by performing Fast Fourier Transforms (FFT) on time-lapsed snapshots taken in the optical microscope. Figure 2h shows that alignment at longer elapsed times is clearly noticed by the formation of an elongated pattern in the FFT images. Alignment was quantified by measuring the aspect ratio of the elongated patterns as a function of time (Fig. 2g). We find that platelets orient parallel to the plane of the rotating magnetic field within the first 60 s of the experiment. Assuming that the normalized aspect ratio of the FFT pattern is proportional to the concentration of fully aligned platelets, one can infer that ∼15 s are required to orient at least half of the suspended anisotropic particles. Since the alignment time depends on the size of the magnetized particles, the wide shape of the alignment curve likely reflects the broad size distribution of platelets used in the study. The experimentally determined alignment time was compared with a theoretical estimate obtained from a balance of torques acting on the platelet's edge (see Supplementary Methods and Supplementary Table 3). Using the average diameter of the platelets in the calculations, we estimate an average alignment time of 5–6 s. Slight platelet agglomeration and unfavourable steric interactions between platelets and the glass slides used in the alignment experiments are not accounted for in this simple calculation, which is probably the reason for the underestimated value obtained. We conclude that the simple analysis proposed can be used as a first order of magnitude estimate of the timescales associated with the texturing process of printed objects. Further investigation of the role of particle agglomeration and steric effects is required to enable a more accurate determination of the texturing timescales.

### Printing of objects with exquisite microstructures

**Figure 3:**
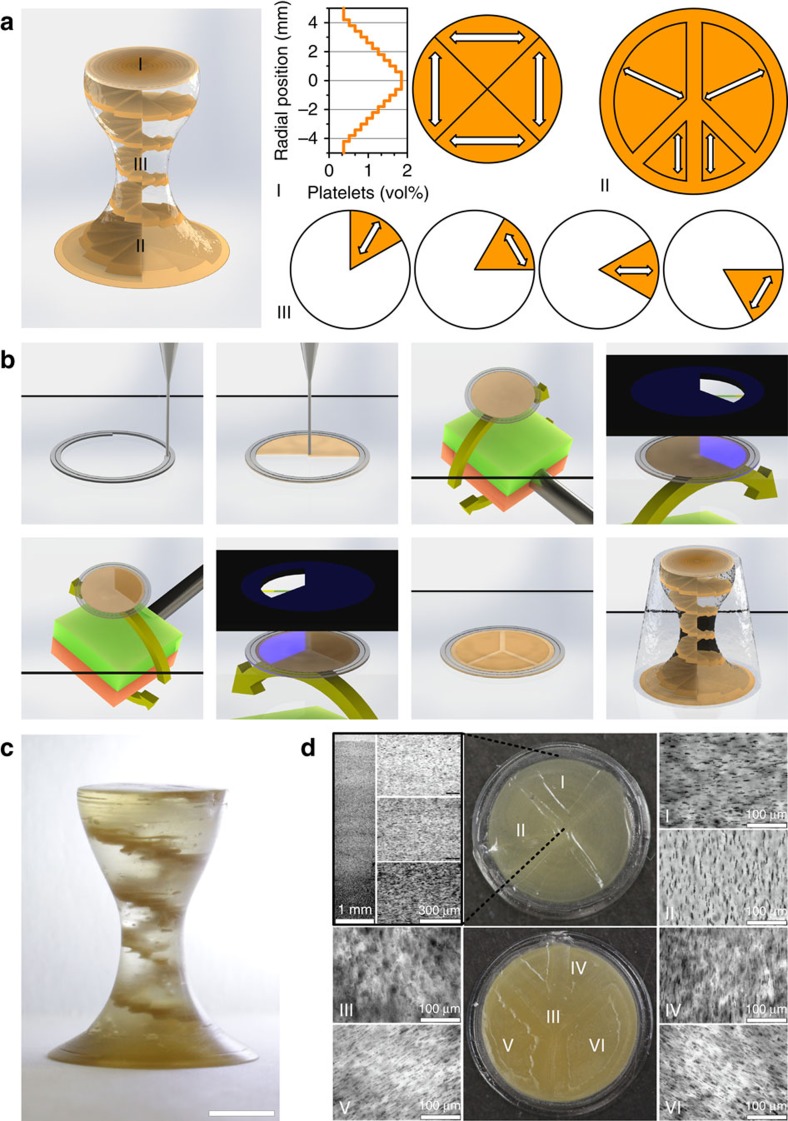
MM-3D printing of complex heterogeneous composite with intricate shape, local texture and local composition. (**a**) Design and programming of the heterogeneous composite, highlighting the changes in the local texture (white arrows) and platelet concentration within the 3D structure. (**b**) Steps involved in the MM-3D printing process. The shaping ink is indicated in grey, whereas the texturing ink is shown in beige. Patterned black foils represent the lithographic masks. (**c**) Actual MM-3D printed object with internal helicoidal staircase. Scale bar, 5mm. (**d**; Top) Photograph of the top layer of the structure, highlighting the successful realization of the programmed gradient in platelet concentration and locally different platelet alignment. Optical micrographs I and II indicate that the local platelet orientation matches the programmed architecture. (**d**; Bottom) Photograph of the bottom layer of the structure, indicating the effective printing of domains with different local platelet orientations: in-plane oriented platelets (III) and out-of-plane oriented platelets aligned along different pre-programmed directions (IV - VI).

To demonstrate our ability to achieve orientation, composition and shape control using the proposed manufacturing platform, we printed an exemplary 3D object with complex geometry and intricate heterogeneous microstructure (Fig. 3a). Two separate inks with distinct rheological behaviour are utilized, namely a viscoelastic ‘shaping ink' and a low-viscosity ‘texturing ink'. The shaping ink is designed to avoid shape distortions due to capillary effects, whereas the texturing ink is formulated to enable alignment of suspended anisotropic particles using a low external magnetic field. The geometry of the object (hereafter named helix) is designed to include both convex and concave curvatures and macroscopic dimensions in the centimetre range. The internal microstructure is programmed to show several heterogeneous features through local tailoring of the orientation and spatial distribution of anisotropic particles and the monomeric composition of the ink. The interior of the object contains locally concentrated platelets in the form of a spiral staircase that spans from the bottom to the top and smoothly conforms to the concave and convex outer surfaces of the 3D object. The local orientation of platelets in each of the staircase steps of the helix is designed to display tangential or radial orientations in an alternating manner from the bottom to the top. The bottom surface of the object is programmed to exhibit four specific areas with three different out-of-plane platelet orientations, which are separated by in-plane-oriented platelets to render a well-defined motif. Finally, the top surface also exhibits four regions with deliberately oriented platelets. In this case, the controlled texture is combined with a spatial gradient in platelet concentration, which continuously increases from the centre to the edge of the top layer.

Printing of this elaborate heterogeneous structure is realized through the combined deposition of the shaping and the texturing inks (printing parameters are shown in Supplementary Tables 4 and 5). The shaping ink is deposited first to generate an outer rim that will define the contour of the 3D object at that specific height (Fig. 3b). The texturing ink is then printed within the contours set by the shaping ink. The resulting layer is positioned under a rotating magnetic field to orient all the platelets suspended in the texturing ink in one specific direction. Exposure of a particular region of the textured layer to ultraviolet light polymerizes and crosslinks the monomer phase of the ink, eventually fixing the platelet orientation in the illuminated area. Patterned photomasks are used to define the illuminated area and thus enable spatial control over the local texture of the printed material. The magnetic alignment and selective curing steps are sequentially repeated until the desired microstructure of that particular layer is achieved. The fabrication process continues by following the same workflow for every deposited ink layer. In addition to texturing through the magnetic alignment process, gradients in the concentrations of constituents (for example, particles and monomers) are easily generated by mixing different ink formulations in a static mixer just before extrusion through the printer nozzle. In the example shown in Fig. 3, a sacrificial shaping ink was used during printing of the outer rim and readily removed after curing the structure, allowing for the polymerized textured ink to be exposed at the outer surface of the object. Careful post treatment with grinding paper ensures a high surface quality of the object.

The intricate heterogeneous architecture of the resulting 3D object is shown in Fig. 3c. Because of the brown colour of the alumina platelets and the optical transparency of the surrounding platelet-free ink, the spiral staircase is readily visible within the interior of the printed object. To assess the efficacy of the magnetic alignment and ink-mixing processes in creating locally heterogeneous textures and gradients, additional individual top and bottom layers were printed and investigated using light microscopy. Indeed, the micrographs shown in Fig. 3d confirm that the programmed heterogeneous microstructure was effectively realized through the proposed printing approach.

### Functional MM-3D printed objects

To illustrate the potential of this printing technique in generating heterogeneous composite architectures with multiple functionalities, we fabricated soft devices that undergo programmed shape changes in 3D when triggered by an external stimulus that causes expansion or contraction of the composite polymer phase. As an example, we use the uptake or removal of a liquid phase from the printed object to impart such dimensional changes, which typically initiates within minutes. Differential expansion or contraction of an organic matrix is a common mechanism used by plant systems to change shape in response to environmental triggers^27^. Here we capitalize on our ability to tune the mechanical properties of the final printed material via proper ink formulation to programme the desired shape change into the 3D objects. The detailed ink formulations and the resulting mechanical properties of the printed materials are shown in Supplementary Tables 2 and 6. While numerous other functionalities can be envisaged, we provide examples where the shape change of heterogeneous composites is exploited to fabricate soft mechanical fasteners that work through unconventional principles. The proposed concept does not require any chemical bonding and relies solely on the mechanical interlocking between parts driven by a programmed shape change of the fastening system.

**Figure 4:**
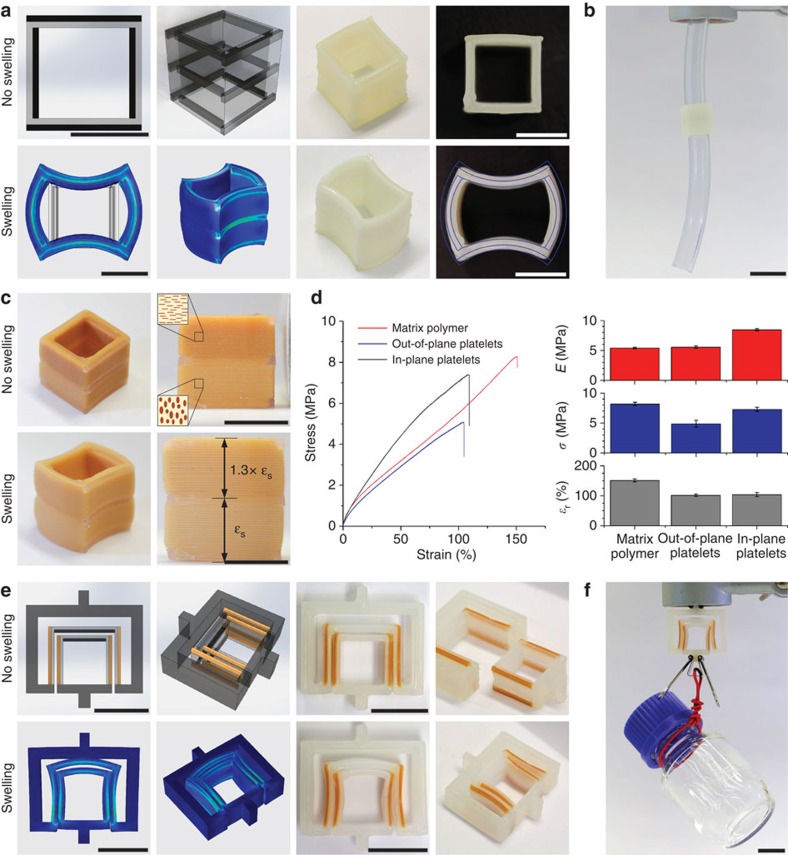
Soft mechanical fasteners fabricated through programmable MM-3D printing of shape-changing objects. (**a**; Top) Drawings and actual printed cuboids before the shape change. This part is named cuboid 1. (**a**; Bottom) Finite element simulations and actual pictures of the cuboids after the shape change. At the bottom right, the simulated cross-section of the cuboid is superimposed on a picture of the actual part to illustrate the good agreement between experimental and predicted curvatures. Scale bars, 10 mm. (**b**) Cuboid-mediated fastening of two tubes achieved through the shape change of the printed object. Scale bar, 20 mm. (**c**) Cuboid object with same architecture as in **a** but containing anisotropic platelets magnetically aligned in different orientations within the soft phase. This part is named cuboid 2. The platelets are aligned horizontally in the top, darker half and vertically in the bottom, brighter half. Scale bar, 10mm. *ɛ*_s_ is the swelling strain. (**d**, Left) Representative stress–strain curves of printed materials comprising the soft phase reinforced with platelets aligned in different orientations. (**d**, Right) Elastic modulus (*E*), strength (*σ*) and strain-at-rupture (*ɛ*_r_) of the printed materials with different textures. Error bars show s.d. (*n*=5). (**e**; Top) Drawings and actual printed key–lock objects before the shape change. (**e**; Bottom) Finite element simulations and actual pictures of the key–lock structure after the shape change. Scale bars, 15 mm. (**f**) Mechanical fastening enabled by the shape-changing key–lock architecture. Scale bar, 15 mm. In all drawings, light and dark grey colours indicate soft and hard phases, respectively.

In the first example, the fastening system consists of a hollow cuboid whose shape change enables joining of two separate elongated parts by a purely mechanical mechanism (Fig. 4a,b). In this system, the internal size of the cuboid is designed to initially match the outer diameter of the elongated parts to be fastened. The matched dimensions enable easy initial sliding and alignment of the parts relative to one another. Fastening occurs by programming the opposing walls of the cuboid to change shape from a flat configuration to concave and convex surfaces. The reduced internal size of the cuboid arising from this shape change compresses the two parts together, fixing them in place by simple mechanical interlocking. This idea was experimentally realized by printing cuboid elements with bilayer walls consisting of crosslinked polymers with different swelling behaviour (Fig. 4a). Remarkably, the cuboid undergoes the expected programmed shape change when allowed to swell in ethyl acetate, which is a good solvent for this particular polymer system. Concave and convex surfaces are generated by inwards and outwards bending of opposing walls of the cuboid. We also performed finite element analysis to simulate the shape change using independently measured swelling and mechanical properties of the crosslinked polymers as input parameters (Supplementary Figs 1 and 2 and Supplementary Table 7). Comparison of the curvature of the concave and convex surfaces of the cuboid object reveals a good agreement between experiments and simulations. From the finite element analysis we predict a curvature of 66 and 74 m^−1^ for the outer surface of the convex and concave walls, respectively. This is close to the experimentally obtained values of 60 and 53 m^−1^ (see Supplementary Table 8). Since this interlocking shape change can in principle be accomplished with water-swellable bioresorbable materials in the absence of any chemical adhesive, the proposed fastening system can be an attractive mechanical means for joining parts in the human body, such as tendons and muscles.

To demonstrate our ability to exploit multimateriality and texturing as two additional independently controlled dimensions for the design of multifunctional 3D printed objects, a second cuboid object was fabricated using similar stiff and soft inks but now including magnetized platelets in one of the inks. In this case, the differential swelling required for the shape change is achieved by combined multimaterials and texturing of the soft polymer ink. In addition, texturing is exploited to independently also control the local mechanical properties of the walls of the object in the axial direction. This is accomplished by aligning platelets horizontally in the top half and vertically in the bottom part of the cuboid (Fig. 4c). The use of a magnet is the key to enable the biaxial alignment of platelets required to maximize their reinforcing effect. Such a magnetic alignment ensures deliberate texture control, which is in strong contrast to the more restrictive shear-induced orientation of anisotropic particles in the printing direction^23^,^28^. Mechanical testing of composites made from such texturing inks showed that the magnetic alignment of 15 wt% (4.4 vol%) of platelets in the tensile loading direction increases the strength and elastic modulus by 49% and 52%, respectively, as compared with specimens with platelets aligned perpendicular to loading (Fig. 4d, Supplementary Fig. 3 and Supplementary Table 6). Texturing was also found to affect the local swelling response of the walls of the printed object, as evidenced by the 30% increase in swelling strain along the *z* direction for walls containing in-plane aligned platelets in comparison with the out-of-plane counterpart (Fig. 4c, Supplementary Fig. 4). Our ability to control the extent of swelling and the mechanical properties of the object in the *z* direction independently from the shape-changing effect illustrates the additional degrees of freedom accessible through MM-3D printing. The effect of texturing on the swelling and mechanical properties of the printed objects can be potentially further amplified by increasing the volume fraction of reinforcing particles in the inks. On the basis of our earlier work on suspensions loaded with magnetized platelets^29^, the volume fraction of platelets in the ink can reach up to 27 vol% if mechanical vibration is provided to improve platelet packing and if the polymerization conditions are optimized to ensure complete monomer conversion in the presence of the light-absorbing iron oxide nanoparticles. Although this example demonstrates well the potential of such multidimensional design space, further systematic studies are required to investigate the extent of reinforcement and shape-changing capabilities that can be covered by each one of these additional printing dimensions.

Besides simple mechanical fastening of parts, shape-changing 3D objects were also printed in complementary geometries to generate smart key–lock connectors that are reconfigurable under external stimuli (Fig. 4e,f). This concept further extends the functionalities of conventional rigid key–lock parts, since it allows for the configuration of multiple key–lock states using the same pair of complementary objects. For example, two soft parts can be designed to first enable easy shape recognition through complementary geometries and later undergo an externally triggered autonomous change in shape to establish a stronger attachment between the key–lock objects. We experimentally realized this design by printing the soft key–lock parts shown in Fig. 4e. The walls of the parts consist of bilayers programmed to undergo complementary shape changes on swelling in a common solvent. The key forms a concave surface of well-defined curvature by the inwards bending of the walls. To ensure the desired geometrical matching, the bilayer walls of the lock are made of the same materials but in a reverse order to generate a convex surface of same curvature through outwards bending. In contrast to the purely frictional forces generated by the cuboid connectors, the reconfigured shape of the key–lock system leads to a geometrically interlocked joint that can withstand much greater mechanical loads. This is demonstrated in Fig. 4f by using the soft fastener to carry a rigid object much heavier than its own weight. Because higher stress concentration occurs at the contact points between mechanically loaded key and lock parts, we utilized in this design composite inks that enable selective reinforcement of the shape-changing walls through the magnetic alignment of reinforcing particles. In addition to mechanical reinforcement, the thermal, optical, magnetic and electrical properties of the walls can be tuned through the deliberate alignment of anisotropic particles in different directions without impairing the targeted shape-changing effect.

## Discussion

Although this work focuses on one particular set of materials, the shape change required for reconfiguration can also be achieved using a much wider range of chemistries and external triggering stimuli. The three-dimensionality of the objects before the shape change also contrasts with the foil or stripe geometries thus far employed to fabricate reconfigurable matter^30^,^31^,^32^,^33^,^34^,^35^,^36^,^37^,^38^. This makes MM-3D printed shape-changing objects much closer to the intricate geometry of plant systems, opening new opportunities for the design and programming of more complex dynamic material systems^19^. Reconfigurable key–lock connectors based on shape-changing parts can potentially be used as autonomously triggered flexible joints, soft building blocks with reversible click-type links as well as selective pick-and-place systems in soft robotics.

The wide design space offered by the proposed MM-3D printing platform greatly expands the current set of toolboxes available for the design and fabrication of functional parts through additive manufacturing technology. Further exploration of such fabrication capabilities will also allow us to more closely replicate in synthetic systems some of the unique microstructural features exhibited by biological materials. Using biological design principles as the guidelines within such a broad parameter space will likely accelerate the development of a new generation of smart composite materials with unparalleled properties and functionalities using more biocompatible, abundant and environmental-friendly resources.

## Methods

### Description of MM-3D printed objects

The 3D objects printed in this study were fabricated using the following workflow: STL data generated in a commercial CAD programme were sliced using the Slic3r software^39^ offered by Alessandro Ranellucci. The resulting G-code was further modified by a customized Python script and later printed using a commercially available 3D printer (3D Discovery, regenHU Ltd, Switzerland).

The helix-like object shown in Fig. 3 is 18 mm high with a circular base of 16 mm diameter and a circular top of 10 mm. The object consists of 60 circular layers, each of which displays a 60°-wide domain printed with a platelet-containing texturing ink (Fig. 3a). The 60°-wide domain is deliberately shifted by 15° in every layer producing a 3D helical stair pattern. The remaining material of the object is clear and without platelets. The platelets in every 60°-wide domain are oriented in either a radial or tangential manner, alternating from one layer to another. The bottom layer was entirely printed with a platelet-containing formulation and contains five domains with three different out-of-plane orientations of platelets (Fig. 3a). The local orientation of the platelets is indicated by white arrows. The platelets outside these domains are oriented in plane. The top layer exhibits a radial gradient in platelet concentration from the centre towards the edge. In addition to this gradient, the layer is divided into four domains with distinct alignment patterns (white arrows in Fig. 3a). The platelet concentration within the gradient varies from ∼1.85 vol% in the centre to 0.37 vol% at the edge. Such a gradient was achieved by mixing a platelet-loaded stem suspension with a pure resin ink in the dual-component dispenser at a volume ratio range varying from 5:1 to 1:5. The helix object was surrounded by a sacrificial shaping ink during printing to allow for shape retention of the platelet-texturing ink and to facilitate printing of the concave shape of the part. However, the results shown in Fig. 2 prove that it is possible to create overhangs and concave geometries using a shaping ink of sufficiently high yield stress, which significantly reduces the printing time.

The cuboids shown in Fig. 4a,c are 15.3 mm high with side lengths of 15 mm. The wall thickness was set to 1.6 mm, which corresponds to twice the printing line width of 0.8 mm. The cuboids consist of two materials, a soft highly swellable polymer and a stiff polymer, as indicated in Supplementary Table 2. For the cuboid 2, platelets were incorporated in the soft phase and were oriented out of plane within the lower half of the object. The upper part was printed with platelets aligned in the printing plane.

Bar-like rigid elements made out of the stiff polymer were incorporated at three positions along the height of the cuboid: the top, the bottom and in the middle (Fig. 4). These bars are 1.5 mm high and 0.8 mm thick. Cuboid 2 was printed with a surrounding sacrificial shaping ink layer to enable retention of the platelet-containing texturing ink, while cuboid 1 was printed without the use of a sacrificial ink.

The fastener shown in Fig. 4e,f is 8.4 mm high and consists of two pieces exhibiting complementary key–lock geometries. Each piece displays a cuboid that allows for a shape-changing effect in part of the object. To achieve shape change, the pieces contain stiff elements that induce bending similar to the cuboid geometry. As opposed to the cuboids, one of the faces is entirely made of stiff polymer to work as attachment point of the fastener. In this case, three materials were incorporated: (1) stiff polymers for all the attachment structures and the stiff bars on the top of the shape-changing cuboid parts (shown in dark grey in Fig. 4e), (2) a soft material for the remainder of the shape-changing cuboid structures (shown in light grey in Fig. 4e) and (3) a platelet-reinforced stiff material for the reinforcement bars on the side of the cuboid structures (shown in brown in Fig. 4e). The platelets were oriented normal to the printing plane and in the axial direction of the bars. Both pieces of the fastener were printed simultaneously, separated by a layer of sacrificial shaping ink. This ink was also applied around all faces containing the texturing ink.

### Formulation and preparation of inks

The ink formulations contained the following main components: PUA oligomers, reactive diluents, photoinitiator, rheology modifier and the alumina platelets. The suppliers of these components and of other chemicals used in this work are shown in Supplementary Table 1. The exact compositions of the formulated inks are shown in Supplementary Table 2. The concentrations of the individual components of the inks were varied to fulfil the specific functionalities desired in each of the printed objects. The mechanical properties of materials printed with such ink formulations are discussed in the Supplementary Methods. PUA oligomers were used as base constituents of all the inks, two of which lead to hard polymers (BR 302 and BR 571) and one of which generates a soft polymer (BR 3641 AJ). The diluents were further added to change the rheological properties of the inks and the mechanical properties of the resulting polymers. Depending on the chemistry of the inks, different grades of FS were used. The hydrophilic grade HDK T30 was applied for more hydrophobic compositions, whereas the more hydrophobic grade HDK H18 was used with more hydrophilic formulations. This enables the creation of a percolating network of silica particles that makes the ink viscoelastic (Fig. 2). For the photoinitiation, we used either Irgacure 907 (BASF, Germany) with ultraviolet light or Irgacure 819 (BASF, Germany) with longer wavelength blue LED light.

The inks were prepared by first dissolving the oligomers in the diluents, followed by the addition of platelets and of the photoinitiator. FS was added to the resulting suspension, which was then homogenized for 5 min at 2,000 r.p.m. in a planetary mixer (ARE-250, Thinky). A degasing step at 2,200 r.p.m. for 5 min was also applied after homogenization. If the ink contained platelets, the mixture was additionally mechanically stirred with a high shear mixer (R 1303 Dissolver stirrer, IKA-Werke GmbH) at 2,000 r.p.m. for 15 min. This de-agglomeration step was also followed by a degasing step. The inks were transferred to 10-cm^3^ printing cartridges and subsequently degased again in the planetary mixer (2,200 r.p.m., 5 min).

### MM-3D printing set-up and protocol

The printing set-up is based on a commercially available direct ink-writing system (3D Discovery, regenHU Ltd). The base system consists of four pressure-driven syringes for the deposition of four different materials. This commercial 3D printer was equipped with a two-component dosing unit (Preeflow EcoDuo450, ViscoTec, Germany) to allow for printing of distinct gradients from tunable mixtures of two different inks. Mixing was performed with the help of a static disposable mixer (MLT 2.5-10-D, Sulzer Mixpac AG, Switzerland).

Alignment and curing were performed using two different magnetic and optical set-ups. In the first magnetic set-up, a rotating neodymium magnet at a fixed position was utilized. A glass plate with adjustable height allowed us to set the distance between the printed object and the magnet. By changing the orientation of the printed object on the glass plate relative to the magnet, the orientation of the platelets was deliberately controlled. The light guide of a spot curing lamp (Omnicure S1000) was directed towards the glass plate to provide the irradiation necessary for polymerization. A printed sample could therefore be subjected to a magnetic field of a rotating magnet and illuminated with ultraviolet light at the same time. A metal frame was constructed for inserting illumination masks between the printed object and the light source. During the printing process, the sample was moved manually between the printer and the alignment/curing set-up. This configuration was employed for the manufacture of the helix and the cuboid 1.

In a more advanced configuration, the alignment and curing steps were directly integrated in the printing machine. This integrated platform was used to print the cuboid 2 and the fastener samples. A rotating magnet set-up with adjustable rotational axes was installed in front of the 3D printer (Fig. 1a). A blue-light light-emitting diode array with attached nitrogen flow was installed at the back of the printer. The stage with the object was programmed to move between the printing, alignment and curing stations during the printing process. Typically, the process started by keeping the stage in the middle position to enable printing of the first layer. The stage was then moved to the front to allow for platelet alignment under the rotating magnet. After alignment, the stage was finally moved all the way back to the curing station. This sequence was repeated successively until completion of the printing operation.

### Shape change experiments and simulation

To trigger shape change of the cuboid, the sample was initially immersed in ethyl acetate overnight, taken out and left to dry. While in ethyl acetate, the cuboid underwent the shape change shown in Fig. 4. Interestingly, the loss of some non-crosslinked material on immersion in ethyl acetate caused the sample to bend towards the opposite direction after complete drying (not shown). This is caused by the stronger shrinkage of the soft phase after dissolution of the non-crosslinked polymer. On re-immersion in the solvent, the original shape was regained in a couple of minutes. Prolonged immersion overnight led again to the bent shape shown in Fig. 4, confirming the reversibility of the shape-changing effect. Eventually, the bent object was used to connect two PVC tubes that had a diameter smaller than the initial gap size of the cuboid. The shape change design and concept outlined here should be easily extendable to other acrylate-based systems that could be triggered for example by water uptake or removal^40^.

After printing the cuboid 2, the support material was removed using a razor blade and the object was immersed in ethyl acetate for 3.5 h to trigger the programmed shape change. The dimensional change along the object's height was tracked by taking snapshots of the swelling part over time (Supplementary Fig. 4).

The two parts of the fastener shown in Fig. 4 were separated after printing and the support material removed using a razor blade. The two parts were immersed in ethyl acetate for ∼30 min to trigger a shape change of the objects. The morphing parts were eventually assembled through mechanical interlocking and mounted on a stand. A 50-ml glass bottle was attached to the fastener to demonstrate the strong attachment obtained through the cooperative shape change of the internal walls of the key–lock matching parts.

Using the data shown in Supplementary Table 7 as input parameters, we performed finite element analyses in COMSOL Multiphysics 5.0 to simulate the shape change of the printed cuboid 1. The constituent materials were modelled as linear elastic solids using the hygroscopic swelling module of the software. Hygroscopic swelling coefficients (*β*) and solvent mass concentrations (*C*) were arbitrarily set to result in the swelling strains (*ɛ*) depicted in Supplementary Table 7, utilizing the relation *ɛ*=*βC*.

### Ink rheology

For the rheological measurements, a base solution of 50 wt% HEMA and 50 wt% PUA BR 571 was prepared and mixed with FS at concentrations ranging from 0 to 8 wt%. All experiments were conducted in a Bohlin Instruments rheometer (Gemini 200 Advanced Rheometer, Software Bohlin V.6.32). Coaxial cylinders with a gap size of 150 μm were used in all the rheological experiments. No pre-conditioning shearing was applied to the tested inks. Storage and loss modulus were obtained from stress-controlled oscillatory measurements performed at 1 Hz and at ambient temperature. In this analysis, the applied stress was increased in a stepwise manner until values were well above the yield stress of the material. The yield stress of the different ink formulations was taken as the crossover points of the storage and loss moduli. The steady-shear viscosity of the inks at distinct shear rates was measured in another experimental series, in which the shear rate was increased stepwise up to ∼1,000 s^−1^.

### 3D shape retention experiments

Printing 3D objects with complex shapes requires the use of inks that are stable against capillary-driven distortions. To evaluate the effect of capillarity on the final shape of deposited inks, we printed films with well-defined initial dimensions and observed changes in their edge curvature after an equilibration time of 10 min. A well-defined initial radius was created by printing an overhanging layer on top of two previously deposited ink layers (Fig. 2d). These experiments were performed using a base ink of HEMA and PUA BR571 (50:50) containing 0.1 wt% Irgacure 819 and FS concentrations ranging from 4 to 8 wt%. The two-component mixer dispenser was used for this experiment to enable more accurate volumetric control of the deposition rate. In this case, both compartments of the dispenser are filled with the same ink material. Three layers with dimensions of 20 × 4 × 0.3 mm were printed. The uppermost layer was shifted to generate an overhang with well-defined offsets of 200 or 400 μm with respect to the layer below. The printing velocity was set to 10 mm s^−1^ and the extrusion rate to 80 μl min^−1^. A conical extrusion tip with a diameter of 410 μm was used. Before polymerization, the samples were equilibrated for 10 min to eliminate distortion effects possibly arising from the printing process. The samples were cured for 60 s with an Omnicure S1000 Mercury lamp under N_2_ atmosphere. Samples were cut in the middle with a razor blade and images of the cross-section were taken with a light microscope. Radius analysis was performed in the middle of the top layer and at the offsets with the software Fiji (Fig. 2d).

### Platelet alignment dynamics

Alignment experiments were performed with a resin formulation consisting of 69.3 wt% HEMA, 29.7 wt% PUA BR-571 and 1 wt% magnetized platelets. A drop of such texturing ink was squeezed between two glass slides and observed under an inverted optical microscope while being subjected to a rotating external magnetic field. A rare earth magnet was positioned in close vicinity to the glass slides at a fixed distance from the ink. A constant magnetic field strength of 40 mT at a rotating speed of 500 r.p.m. was applied. A sequence of images was taken every 30 s for 10 min to monitor the degree of platelet alignment as a function of time. To quantify the degree of alignment, the obtained images were rendered binary and a FFT was performed using the software Fiji^41^. The vertical and the horizontal diameters of the resulting pattern were used for alignment quantification. For a first-order approximation, such diameters were taken as the distances at full-width at half-maximum of Gaussian fits to the brightness distributions on both axes. The ratio between these two diameters were then used as the alignment factor shown in Fig. 2g.

## Additional information

**How to cite this article:** Kokkinis, D. *et al.* Multimaterial magnetically assisted 3D printing of composite materials. *Nat. Commun.* 6:8643 doi: 10.1038/ncomms9643 (2015).

## Supplementary Material

Supplementary Information Supplementary Figures 1-4, Supplementary Tables 1-8, Supplementary Methods and Supplementary References

## References

[b1] Sachs E., Cima M., Williams P., Brancazio D. & Cornie J. 3-Dimensional printing—rapid tooling and prototypes directly from a CAD model. ASME J. Eng. Ind. T 114, 481–488 (1992) .

[b2] Kodama H. Automatic method for fabricating a 3-dimensional plastic model with photo-hardening polymer. Rev. Sci. Instrum. 52, 1770–1773 (1981) .

[b3] Villar G., Graham A. D. & Bayley H. A tissue-like printed material. Science 340, 48–52 (2013) . 23559243 10.1126/science.1229495 PMC3750497

[b4] Fantini M., de Crescenzio F., Persiani F., Benazzi S. & Gruppioni G. 3D restitution, restoration and prototyping of a medieval damaged skull. Rapid Prototyping J. 14, 318–324 (2008) .

[b5] Oxman N., Tsai E. & Firstenberg M. Digital anisotropy: a variable elasticity rapid prototyping platform. Virtual Phys. Prototyp. 7, 261–274 (2012) .

[b6] Dimas L. S., Bratzel G. H., Eylon I. & Buehler M. J. Tough composites inspired by mineralized natural materials: computation, 3d printing, and testing. Adv. Funct. Mater. 23, 4629–4638 (2013) .

[b7] Sun K. *et al.* 3D printing of interdigitated li-ion microbattery architectures. Adv. Mater. 25, 4539–4543 (2013) . 23776158 10.1002/adma.201301036

[b8] Murphy S. V. & Atala A. 3D bioprinting of tissues and organs. Nat. Biotechnol. 32, 773–785 (2014) . 25093879 10.1038/nbt.2958

[b9] Fu Q., Saiz E. & Tomsia A. P. Direct ink writing of highly porous and strong glass scaffolds for load-bearing bone defects repair and regeneration. Acta Biomater. 7, 3547–3554 (2011) . 21745606 10.1016/j.actbio.2011.06.030 PMC3163833

[b10] Rudykh S., Ortiz C. & Boyce M. C. Flexibility and protection by design: imbricated hybrid microstructures of bio-inspired armor. Soft Matter 11, 2547–2554 (2015) . 25715866 10.1039/c4sm02907k

[b11] Ge Q., Qi H. J. & Dunn M. L. Active materials by four-dimension printing. Appl. Phys. Lett. 103, 131901 (2013) .

[b12] Tibbits S. 4d printing: multi-material shape change. Archit. Design 84, 116–121 (2014) .

[b13] Raviv D. *et al.* Active printed materials for complex self-evolving deformations. Sci. Rep. 4, 7422 (2014) . 25522053 10.1038/srep07422 PMC4270353

[b14] Hardin J. O., Ober T. J., Valentine A. D. & Lewis J. A. Microfluidic printheads for multimaterial 3D printing of viscoelastic inks. Adv. Mater. 27, 3279–3284 (2015) . 25885762 10.1002/adma.201500222

[b15] Bakarich S. E., Gorkin R., Panhuis M. I. H. & Spinks G. M. 4D printing with mechanically robust, thermally actuating hydrogels. Macromol. Rapid Commun. 36, 1211–1217 (2015) . 25864515 10.1002/marc.201500079

[b16] Yu K., Ritchie A., Mao Y., Dunn M. L. & Qi H. J. Controlled sequential shape changing components by 3D printing of shape memory polymer multimaterials. Procedia IUTAM 12, 193–203 (2015) .

[b17] Guiducci L., Weaver J. C., Brechet Y. J. M., Fratzl P. & Dunlop J. W. C. The geometric design and fabrication of actuating cellular structures. Adv. Mater. Interfaces 2,, doi:10.1002/admi.201500011 (2015) .

[b18] Cooper G. M. The Cell—A Molecular Approach 2nd edn Sinauer Associates (2000) .

[b19] Studart A. R. Biologically inspired dynamic material systems. Angew. Chem. Int. Ed. Engl. 54, 3400–3416 (2015) . 25583299 10.1002/anie.201410139

[b20] Studart A. R. Biological and bioinspired composites with spatially tunable heterogeneous architectures. Adv. Funct. Mater. 23, 4423–4436 (2013) .

[b21] Dunlop J. W. C. & Fratzl P. Biological composites. Annu. Rev. Mater. Res. 40, 1–24 (2010) .

[b22] Tekinalp H. L. *et al.* Highly oriented carbon fiber-polymer composites via additive manufacturing. Composites Sci. Technol. 105, 144–150 (2014) .

[b23] Compton B. G. & Lewis J. A. 3D-printing of lightweight cellular composites. Adv. Mater. 26, 5930–5935 (2014) . 24942232 10.1002/adma.201401804

[b24] Calvert P. & Crockett R. Chemical solid free-form fabrication: making shapes without molds. Chem. Mater. 9, 650–663 (1997) .

[b25] Erb R. M., Libanori R., Rothfuchs N. & Studart A. R. Composites reinforced in three dimensions by using low magnetic fields. Science 335, 199–204 (2012) . 22246772 10.1126/science.1210822

[b26] Erb R. M., Segmehl J., Charilaou M., Loffler J. F. & Studart A. R. Non-linear alignment dynamics in suspensions of platelets under rotating magnetic fields. Soft Matter 8, 7604–7609 (2012) .

[b27] Burgert I. & Fratzl P. Actuation systems in plants as prototypes for bioinspired devices. Phil. Trans. R Soc. A 367, 1541–1557 (2009) . 19324722 10.1098/rsta.2009.0003

[b28] Martin J. J., Riederer M. S., Krebs M. D. & Erb R. M. Understanding and overcoming shear alignment of fibers during extrusion. Soft Matter 11, 400–405 (2015) . 25408494 10.1039/c4sm02108h

[b29] Libanori R., Erb R. M. & Studart A. R. Mechanics of platelet-reinforced composites assembled using mechanical and magnetic stimuli. ACS Appl. Mater. Interfaces 5, 10794–10805 (2013) . 24102294 10.1021/am402975a

[b30] Erb R. M., Sander J. S., Grisch R. & Studart A. R. Self-shaping composites with programmable bioinspired microstructures. Nat. Commun. 4, 1712 (2013) . 23591879 10.1038/ncomms2666

[b31] Gracias D. H., Kavthekar V., Love J. C., Paul K. E. & Whitesides G. M. Fabrication of micrometer-scale, patterned polyhedra by self-assembly. Adv. Mater. 14, 235–238 (2002) .

[b32] Smela E., Inganas O. & Lundstrom I. Controlled folding of micrometer-size structures. Science 268, 1735–1738 (1995) . 17834992 10.1126/science.268.5218.1735

[b33] Ionov L. Soft microorigami: self-folding polymer films. Soft Matter 7, 6786–6791 (2011) .

[b34] Hawkes E. *et al.* Programmable matter by folding. Proc. Natl Acad. Sci. USA 107, 12441–12445 (2010) . 20616049 10.1073/pnas.0914069107 PMC2906559

[b35] Ahn B. Y. *et al.* Printed origami structures. Adv. Mater. 22, 2251–2254 (2010) . 20397151 10.1002/adma.200904232

[b36] Gracias D. H. Stimuli responsive self-folding using thin polymer films. Curr. Opin. Chem. Eng. 2, 112–119 (2013) .

[b37] Leong T. G., Zarafshar A. M. & Gracias D. H. Three-dimensional fabrication at small size scales. Small 6, 792–806 (2010) . 20349446 10.1002/smll.200901704 PMC3078552

[b38] Judy J. W. & Muller R. S. Magnetically actuated, addressable microstructures. J. Microelectromech. Syst. 6, 249–256 (1997) .

[b39] Ranellucci A. *Slic3r- G-code generator for 3D printers* <http://www.slic3r.org/> (2015) .

[b40] Baudis S. *et al.* Photopolymerizable elastomers for vascular tissue regeneration. J. Macromol. Syst. 296, 121–126 (2010) .

[b41] Gunes D. Z., Scirocco R., Mewis J. & Vermant J. Flow-induced orientation of non-spherical particles: Effect of aspect ratio and medium rheology. J. Non.-Newton. Fluid 155, 39–50 (2008) .

