# Supplementary material for: Multimaterial magnetically assisted 3D printing of composite materials
Source: Nat Commun. 2015 Oct 23;6:8643. doi: 10.1038/ncomms9643 (PMC4639895; doi:10.1038/ncomms9643)
Supplement: Supplementary Information — Supplementary Figures 1-4, Supplementary Tables 1-8, Supplementary Methods and Supplementary References [file ncomms9643-s1.pdf]

Supplementary Information

Supplementary Figures

Stiff material de-swelled	Stiff material swollen	Soft material de-swelled	Soft material swollen

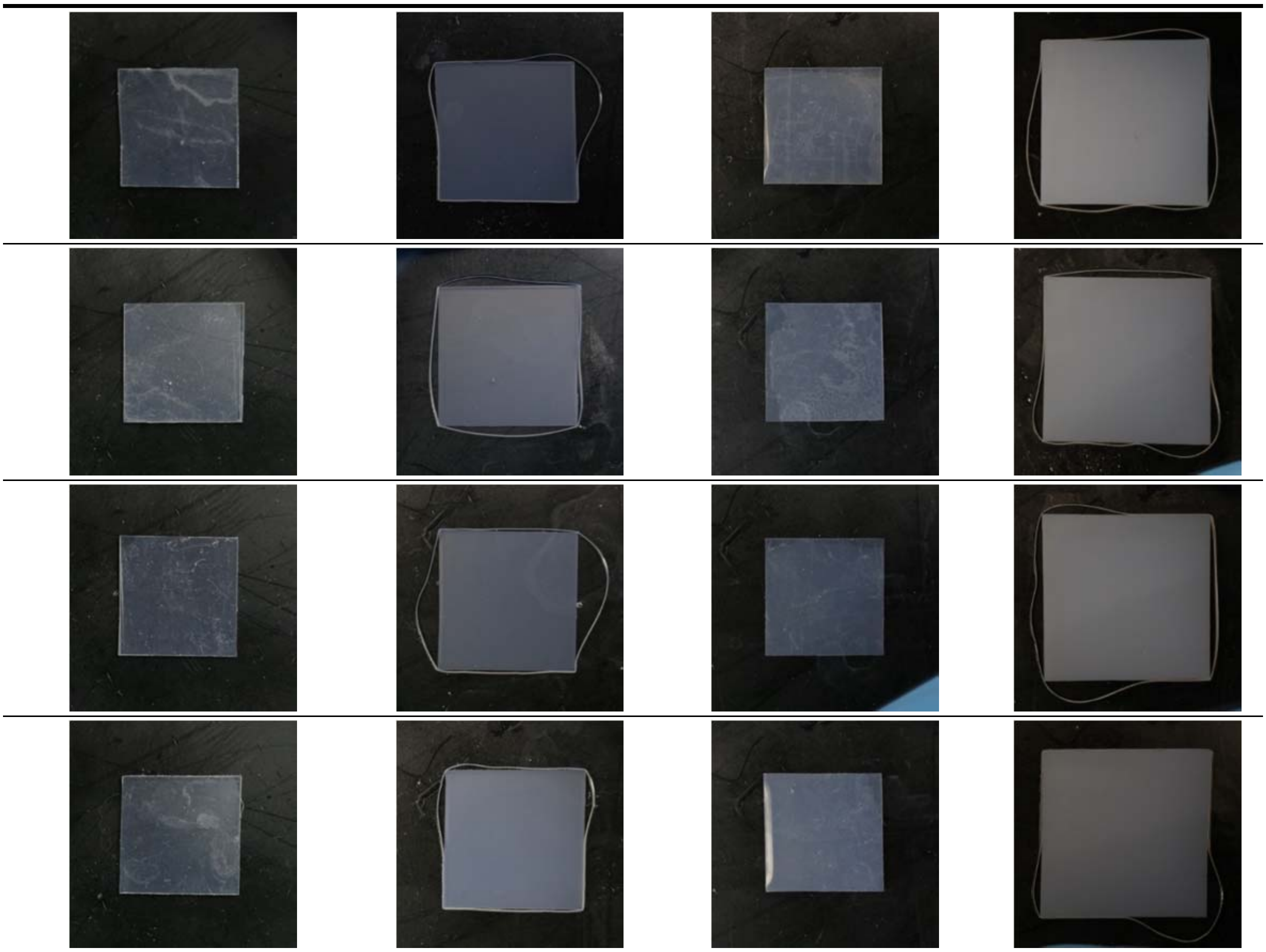

Supplementary Figure 1: Photographs of polymer films before and after swelling in ethyl acetate. Each row shows an independent specimen of a total of four used to obtain an average value for the swelling strains.

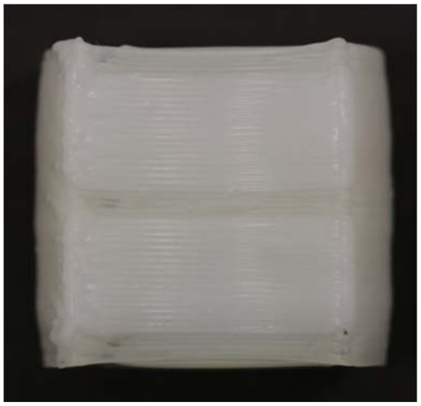

Supplementary Figure 2: Photographs of the cuboid 1 object after swelling in ethyl acetate.

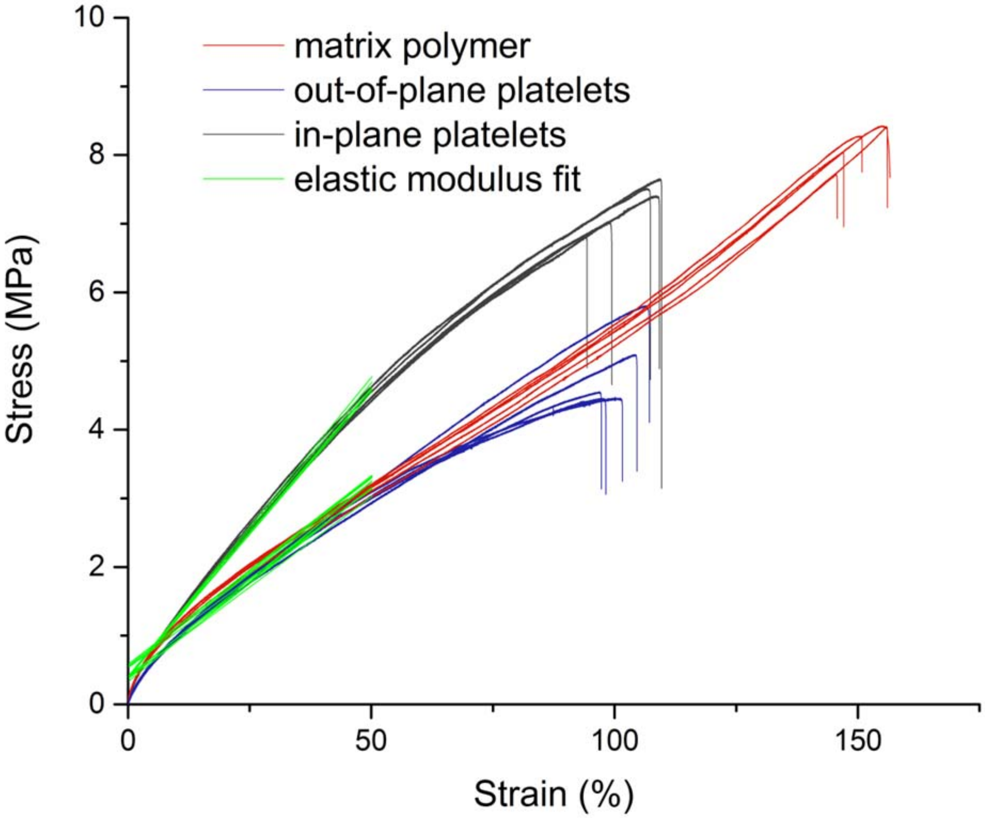

Supplementary Figure 3: Stress-strain data obtained from tensile tests of materials made from the texturing ink containing platelets with different orientations. Data was compared to results measured for the polymer matrix alone.

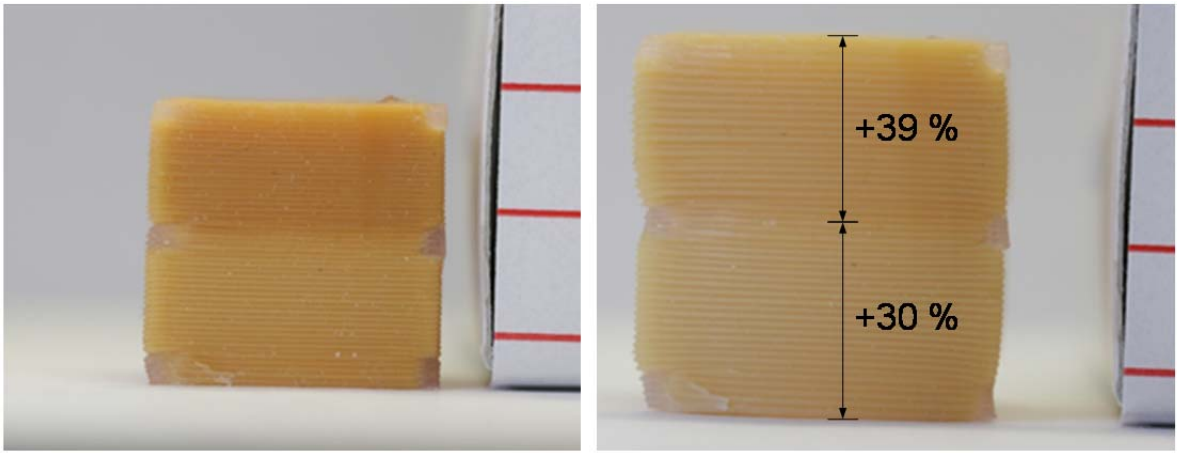

Supplementary Figure 4: Photographs of the cuboid 2 object. Images were taken before (left) and after (right) swelling the object in ethyl acetate. Swelling strains of 39 and 30% were measured for the top and for the bottom parts, respectively.

Supplementary tables

Supplementary Table 1: Chemicals and consumables used in this work.

Name	Abbreviation	Brand name	Supplier
Difunctional aliphatic urethane acrylate		Bomar BR-571	Dymax Oligomers and Coatings
Aromatic difunctional urethane acrylate oligomer		Bomar BR-302	Dymax Oligomers and Coatings
1.3 Functional aliphatic urethane acrylate oligomer		Bomar BR-3641 AJ	Dymax Oligomers and Coatings
2-Hydroxyethyl methacrylate; 97%	HEMA		ABCR-Chemicals
Isodecyl acrylate	IDA		Sigma-Aldrich
Isobornyl acrylate	IBOA	SR506D	Sartomer
Pentaerythritol tetrakis(3-mercaptopropionate) >95%	PTM		Sigma-Aldrich
Hydrophilic pyrogenic silica		HDK T30	Wacker Chemie AG
Hydrophobic pyrogenic silica		HDK H18	Wacker Chemie AG
Bis(2,4,6-trimethylbenzoyl)-phenylphosphineoxide		Irgacure 819	BASF
2-Methyl-1[4-(methylthio)phenyl]-2-morpholinopropan-1-one		Irgacure 907	BASF
Alumina platelets		RonaFlair White Sapphire	Merck
Ferrofluid		EMG 705	Ferrotec
Static 2 component mixer		MLT 2.5-10-D	Sulzer Mixpac AG
Glass hydrophobisation treatment		Rain-X water repellant	Rain-X

Supplementary Table 2: Ink formulations used in this work (wt%).

	PUA oligomers			Reactive diluents				Rheology modifier		Photoinitiator		
	**BR 302**	**BR 571**	**BR 3641 AJ**	**HEMA**	**IDA**	**IBOA**	**PTM**	**HDK T30**	**HDK H18**	**Irgacure 907**	**Irgacure 819**	**Platelets**
**Helix**												
Shaping ink	-	41.00	-	-	-	50.00	-	7.00	-	2.00	-	-
Texturing ink	-	40.61	-	-	-	49.52	-	-	-	1.98	-	7.89
Sacrificial shaping ink	-	41.84	-	-	-	51.02	-	7.14	-	-	-	-
**Cuboid 1**												
Stiff shaping ink	42.00	-	-	50.00	-	-	-	-	6	2.00	-	-
Soft shaping ink	21.00	-	19.75	25.00	12.50	12.50	0.25	-	7	2.00	-	-
**Cuboid 2**												
Stiff shaping ink	-	45.75	-	45.75	-	-	-	-	8	-	0.5	-
Soft texturing ink	19.29	-	18.54	22.96	11.74	11.74	0.23	-	-	-	0.5	15
Sacrificial shaping ink	10.50	-	30.28	12.50	19.17	19.17	0.38	-	8	-	-	-
**Key-lock fastener**												
Stiff shaping ink	-	45.75	-	45.75	-	-	-	-	8	-	0.5	-
Soft shaping ink	20.89	-	20.08	24.86	12.71	12.71	0.25	-	8	-	0.5	-
Stiff texturing ink		42.25	-	42.25	-	-	-	-	-	-	0.5	15
Sacrificial shaping ink	21.00	-	20.19	25.00	12.78	12.78	0.26	-	8	-	-	-
**Rheology, Shaping and Alignment**												
Rheology	-	46-50	-	46-50	-	-	-	-	0-8	-	-	-
3D Shaping	-	45.95-47.95	-	45.95-47.95	-	-	-	-	4-8	-	0.1	-
Alignment analysis	-	29.7	-	69.3	-	-	-	-	-	-	-	1

Supplementary Table 3: Input parameters used for the estimation of the time required for alignment of platelets in the presence of an external magnetic field.

Fluid viscosity, η (Pa.s)	1.15
Volume fraction of iron oxide nanoparticles relative to alumina	0.91
Magnetic field (mT)	40
Platelet thickness, 2a (µm)	0.4
Platelet diameter, 2b (µm)	8
Magnetic shell thickness, d (nm)	12
Magnetic permeability of free space, μ_0 (m kg s^{-2} A^{-2})	1.255×10^{-6}
Magnetic susceptibility of the particle shell, χ_{ps}	2.879

Supplementary Table 4: Printing parameters used for the fabrication of the different objects investigated in this study.

	Alignment			Curing				Printing					
	Rotation speed magnet [rpm]	Magnet distance [cm]	Time [min]	Setup	Time [min]	Power	Lamp distance [cm]	Feed speed [mm/s]	Layer height [mm]	Needle diameter [mm]	Line width [mm]	Start delay [s]	Stop delay [s]
**Helix**	500	2.5	5	Omnicure	1	100%	10	10	0.3	0.41 metal/ 0.41 plastic	0.4	1/18-3/18	1
**Cuboid 1**	-	-	-	Omnicure	0.5	100%	10	10	0.3	0.58 metal	0.8	5/18	1
**Cuboid 2**	200	4	2	2x440nm 1x405nm	2	1.6 A 3.6V	5	10	0.3	0.58 metal	0.8	5/18	1
**Fastener**	200	4	2	2x440nm 1x405nm	3	1.6 A 3.6V	5	10	0.3	0.58 metal	0.8	5/18	1

Supplementary Table 5 Printing parameters used to fabricate the top layer of the helix object.

Printing parameters				Mixer flushing parameters			
start delay [s]	extrusion speed [µl min^{-1}]	suckback volume [µl]	suckback speed [µl min^{-1}]	extrusion volume [µl]	extrusion speed [µl min^{-1}]	suckback volume [µl]	suckback speed [µl min^{-1}]
21/18	79	2	300	200	500	4	1000

Supplementary Table 6: Mechanical data for materials made from the texturing ink used to fabricate the cuboid 2.

	Elastic modulus (MPa)		Strength (MPa)		Strain at rupture (%)	
	average	SD	average	SD	average	SD
**Polymer matrix**	5.4	0.13	8.2	0.30	151.3	5.0
**Composite with platelets aligned out-of-plane**	5.6	0.21	4.9	0.58	101.8	4.2
**Composite with platelets aligned in-plane**	8.5	0.16	7.3	0.35	104.0	6.8

Supplementary Table 7: Parameters used in the shape change simulation of the cuboid 1 object.

E_{stiff}	6.63	MPa
E_{soft}	2.03	MPa
ε_{stiff}	0.149	-
ε_{soft}	0.377	-
**Density stiff mat**	1.1	g cm^{-3}
**Density soft mat**	1.1	g cm^{-3}
**Poisson's ratio**	0.4	-

Supplementary Table 8: Comparison of the curvature values measured for the swollen cuboid 1 object and the finite element simulation.

	Curvature (m^{-1})			
	experiment		simulation	
	inside	outside	inside	outside
**convex**	68	60	91	66
**concave**	44	53	54	74

Supplementary Methods

Platelet modification and size analysis

The alumina platelets used in this study (Ronaflair WhiteSaphire, Merck) were magnetized as described by Erb *et al.* [1] Typically, 10 g of platelets were dispersed in 200 ml of double deionized water. Then, 375 µL of the ferrofluid EMG 705 (Ferrotec) were dissolved in 20 ml double deionized water and dropped into the alumina suspension. The suspension was left to stir over night, filtered in a Büchner funnel with the aid of vacuum suction and washed with ethanol. The filter cake was dried in the oven at 100°C for at least 2 hours.

The maximum Feret diameter was measured by fitting circles to platelets imaged by SEM using the software Fiji. A total of 337 platelets were used for the diameter analysis, following the protocol described in an earlier publication. [2] The thickness was determined on a cross section of an aligned platelet composite from SEM images kindly supplied by Hortense Le Ferrand. 300 platelets were analyzed for thickness determination. An average diameter of 8.3 ± 4 µm and an average thickness of 403 ± 139 nm were found for these platelets.

Printing protocol

Before every printing procedure, the ink cartridges were equipped with needles and inserted into their respective positions in the printer. xy- and z-calibrations were performed for every needle individually. The pressure-controlled extruded volumes, as well as start-stop delays, were calibrated using a specifically designed printing pattern.

Glass slides were hydrophobised using a commercial surface treatment (Rain-X water repellant, Rain-X) to serve as printing substrates. A feed speed of 10 mm/s was used in all printing procedures. The exact printing parameters and needle diameters can be found in Supplementary Table 4. For the printing of the gradient structures, the two component dispenser mixer unit was used. This unit is equipped with more advanced additional control options, as indicated in Supplementary Table 5. Unlike the pressure controlled syringes, the dual-dispenser enables direct control of the extruded volume. Moreover, after printing each filament, the extruder can be programmed to suck back a minute amount of material with a predefined speed. Using this feature, material dripping and accumulation at stop points is avoided. Due to the finite dead volume of the static mixer component, the dispenser had to be flushed after each change of mixing ratio. This typically occurred before printing a new line with a new composition.

Alignment protocol

In addition to the alignment results reported in Figure 2 for one specific ink (main text), we also conducted experiments to determine the time required for platelet alignment in other ink formulations. To this end, single printed layers were exposed to the rotating magnet for different time periods and the degree of platelet alignment was afterwards assessed under an optical microscope. On the basis of these experiments, we defined the alignment time for each of the printed objects. For the cuboid and fastener samples, the alignment time was set to 2 min at a magnet rotational speed of 200 rpm. The helix sample was printed using an alignment time of 5 min and rotating field of 500 rpm for every layer. These time periods were rather conservative to ensure effective alignment of the platelets.

Curing/masking protocol

The helix and the cuboid 1 samples were cured using an Omnicure UV-lamp (18.5 W cm^{-2} for 320-500 nm). Individual printed layers of the helix and cuboid 1 were illuminated at 100% intensity for 1 min and 30 s, respectively. For the helix sample, the top and bottom layers were programmed to show areas exhibiting different platelet alignment. To cure only specific areas of those samples, we used customized masks made out of paper glued on glass slides for stability and easy manipulation. Also in this case, areas of the layer with different platelet alignment were illuminated for 1 min.

The cuboid 2 and the fastener objects were cured using a different setup. A custom LED array was built using two 440 nm 3 W and one 405 nm 3 W LEDs. The diodes were mounted in a plastic housing with reflective walls. A plastic hose connected to a nitrogen gas supply was attached to the housing. This setup allowed for increased light intensity and a locally enriched nitrogen atmosphere that prevents O_2-deactivation of the initiator present in the ink. Layers of the cuboid 2 and fastener objects were cured under nitrogen flow for 2 min and 3 min, respectively.

Overall, the direct writing, particle alignment and curing steps described above lead to printing times within the range of hours, depending on the number of printed layers. As an example, the cuboid 2 shown in Figure 4 has 51 layers and required a total printing time of 5 hours. In this study, we used the following typical values for the individual steps: 2 min for direct writing, 2 min for alignment and 2 min for light curing. These values are all very conservative and were chosen to ensure complete alignment and monomer conversion. Further optimization of the ink formulation and processing conditions should decrease the total printing time. Parameters that can be further optimized to reduce the printing time include: the light source, the iron oxide content on the platelets, the printing layer thickness or the illumination time of the samples.

Structural characterization of the Helix object

After printing the helix, the support material was removed using a razor blade and the surface of the object was ground using grinding paper before taking the photograph shown in Figure 3. To better visualize the orientation of the platelets, single layers were printed directly on a non-hydrophobized glass slide and investigated by transmission light microscopy. The micrographs showing the gradient in the top layer (Figure 3) were put together from a sequence of different microscopy images that were obtained with the same exposure settings. Prior to taking these images, the sample was ground to reduce effects of topography.

Swelling strains in polymer films for shape change simulation

The expansion behavior of the individual constituent materials of cuboid 1 was first independently measured in homogeneous films prepared with the same ink composition leading to the soft and stiff elements of the object. To this end, 300 μm thick films were cast and polymerized between two glass slides using an Omnicure S1000 mercury lamp for 1 minute under full illumination conditions. Square specimens with a length of 12.7 mm were cut out of these films (Supplementary Fig. 1). These specimens were then immersed in ethyl acetate overnight, dried and swelled for 20 mins, following a procedure similar to that applied to trigger shape changes in the cuboid 1 sample. Photographs were taken as a function of time to determine the dimensional changes induced by swelling of the polymer. Swelling strains were calculated with respect to the initial side length of 12.7 mm before the swelling. The results show a difference of 0.228 in the swelling strain between the stiff and the soft polymers. The strain was found to be 0.377 for the soft phase and 0.149 for the stiff phase.

Swelling strains measured directly in cuboid 1 object

In addition to the independent measurements discussed above, the swelling strains of the soft and stiff phases were also determined by quantifying dimensional changes directly on the cuboid 1 object. Because the 3D sample exhibits complex topography, such direct strain measurements are expected to show limited accuracy. Therefore, this analysis is only intended to verify if the measurements made on individual films provide results in the same order of magnitude of the actual deformations in the cuboid sample. This comparative analysis also allows us to evaluate if the time period used for swelling of the films was sufficiently long to reach the equilibrium state expected after swelling of the more complex cuboid geometry. Supplementary Fig. 2 shows an image of the cuboid sample used for the direct measurements of the swelling-induced strains. The strains were determined to be 0.368 for the soft phase and 0.156 for the stiff phase. The results obtained from such direct measurements agree very well with the data acquired from individual films (see section above).

It is important to note that the direct measurements on the cuboid were performed after immersion overnight in ethyl acetate as opposed to a timeframe of only 20 min used for the swelling of the individual polymer films. We found that 20 min was clearly not enough time to swell the cuboid 1 to an equilibrium state (data not shown), which is probably due to the thicker walls of the object as compared to the cast films. On the basis of the comparable results for the swelling of individual films and the direct measurement on the final object, we conclude that the different swelling times used in the two experimental series was long enough to allow the materials to reach an equilibrium state.

Determination of the elastic modulus for shape change simulation

Besides swelling strains, the elastic modulus of the constituent materials is another important input parameter to simulate the shape change of the cuboid object. To determine this mechanical property, we first printed plates with a thickness of 3 mm using the cuboid 1 materials. Discs with a diameter of 12 mm were then punched out of these plates and soaked in ethyl acetate overnight before mechanical testing. Compression tests were performed at a crossbar speed of 0.5 mm min^{-1} while keeping the specimens completely immersed in ethyl acetate. Samples were loaded up to a force of 50 N. The elastic modulus in compression was calculated by fitting the linear section of the obtained stress-strain curves. The moduli in the swollen state were found to be 2.033 ± 0.05 and 6.633 ± 1.46 MPa for the soft and stiff materials, respectively. This corresponds to a stiffness ratio of 3.27.

Shape change simulations

The simulated geometry of the cuboid after swelling is indicated by the blue lines shown in Figure 4a (main text). The geometry obtained from the simulation is superimposed on an image of the top of the actual cuboid in order to directly compare the finite element analysis with the experimental result. We find that the simulation slightly overestimates the overall dimensions of the object. Very good agreement is obtained for the curvature of the concave and convex surfaces, as indicated in Supplementary Table 8. This result indicates that finite element analysis is an effective tool to predict and program the shape change of MM-3D printed objects.

Based on the good agreement between simulations and experiments for the cuboid object, we used the finite element method to qualitatively predict the shape change of the key-lock fastener (Figure 4c) without knowing a priori the exact mechanical properties of its constituent

materials. Taking the elastic moduli of the soft and stiff polymers utilized for the cuboid sample as a first approximation, we determined the difference in swelling strain between the stiff and the soft phases needed to match the simulated and the experimental curvatures of the shape-changing part of the fastener. Under these conditions, we find that a swelling strain mismatch of 0.1 is required to qualitatively describe the shape change of the fastener. Since this value is within the same order of magnitude of the value of 0.228 experimentally measured for the soft and hard phases of the cuboid, we conclude that the input data shown in Supplementary Table 7 may be used to obtain a first qualitative picture of the shape change to be expected from objects printed from the soft and stiff ink materials developed in this work. A more quantitative prediction requires more accurate determination of the swelling and mechanical input parameters, as shown above for the cuboid objects.

Platelet alignment dynamics theory

The time t required for the alignment of an individual platelet subjected to a rotating magnetic field H in a Newtonian fluid can be theoretically estimated by performing a torque balance at the edge of the platelet, as previously suggested in the literature. [3,4] Considering that platelet rotation occurs when the magnetic torque reaches the same order of magnitude of the viscous torque, one can arrive at the following expression for the alignment time:

$$t = \frac{\pi^2 K_1 \eta}{2 K_2 H^2} \tag{1}$$

where:

η is the viscosity of the Newtonian fluid,

$$K_1 = 6V \frac{f}{f_0} \tag{2}$$

$$K_2 = \frac{2\pi\mu_0 \chi_{ps}^2}{3\left(\chi_{ps}+1\right)}\left[\left(a+d\right)\left(b+d\right)^2 - ab^2\right] \tag{3}$$

V is the volume of the platelet given by $V = 2\pi ab^2$, (4)

a and b are half the thickness and half the diameter of the platelet, respectively;

d is the thickness of the magnetic shell coating the platelets,

f/f_0 is the Perrin friction factor given by $\frac{f}{f_0} = \frac{4\left(1-p^2\right)}{3\left(2-p^2 aS\right)}$, (4)

p is the aspect ratio of the platelets (b/a),

$$S = \left(\frac{2}{a}\right)\left(p^2 - 1\right)^{-1/2} \tan^{-1}\left[\left(p^2 - 1\right)^{1/2}\right], \quad (6)$$

μ_0 is the magnetic permeability of free space,

and χ_{ps} is the magnetic susceptibility of the particle shell.

Equation 1 was used to estimate the time required for the alignment of platelets used in the experiments reported in Figure 2g,h. The input parameters depicted in Supplementary Table 3 were used in these calculations.

Mechanics of platelet-containing texturing ink used in cuboid 2

The mechanical properties of materials obtained from the texturing ink used in cuboid 2 (Supplementary Table 2) were measured in tensile mode to evaluate the effect of different platelet orientations as compared to the pure polymer matrix. To this end, polymer films were printed using the setup described in the method section (main text) for the printing of the cuboid 2. The curing time was set to 5 min instead of 2 min to exclude any possible influence of the stronger light absorption characteristics of iron oxide coated-platelets on the properties of the printed material. Small dogbone samples were cut from the polymer films. Tensile tests were performed by applying a pre-load of 0.05 N, followed by continuous loading at a crosshead speed of 5 mm/min. The elastic modulus was determined by fitting a straight line to the stress-strain data in the strain range of 0.1-50% (Supplementary Fig. 3).

The in-plane alignment of platelets parallel to the loading direction was found to increase the strength and the elastic modulus of the composite by 49% and 52% as compared to specimens with platelets aligned out-of-plane (Supplementary Fig. 3 and Supplementary Table 6). Interestingly, this reinforcement effect of magnetically aligned platelets occurs without any loss in the stretchability of the composite. When compared to the pure polymer matrix, such reinforcement represents a 57% increase of the elastic modulus of the material, upon the incorporation of aligned alumina platelets. Such effect is accompanied by a 31% decrease in the total stretchability and 11% decrease in strength of the material. These results confirm our ability to control the mechanical properties and resulting swelling response of the printed composites by magnetically changing the orientation of reinforcing platelets.

Supplementary References

1 Erb, R. M., Libanori, R., Rothfuchs, N. & Studart, A. R. Composites Reinforced in Three Dimensions by Using Low Magnetic Fields. *Science* **335,** 199-204 (2012).

2 Libanori, R., Erb, R. M. & Studart, A. R. Mechanics of platelet-reinforced composites assembled using mechanical and magnetic stimuli. *ACS Appl. Mater. Interfaces* **5,** 10794-10805 (2013).

3 Erb, R. M., Segmehl, J., Charilaou, M., Loffler, J. F. & Studart, A. R. Non-linear alignment dynamics in suspensions of platelets under rotating magnetic fields. *Soft Matter* **8,** 7604-7609 (2012).

4 Erb, R. M., Segmehl, J., Schaffner, M. & Studart, A. R. Temporal response of magnetically labeled platelets under dynamic magnetic fields. *Soft Matter* **9,** 498-505 (2013).
